# Proteomic-Based Identification of CD4-Interacting Proteins in Human Primary Macrophages

**DOI:** 10.1371/journal.pone.0018690

**Published:** 2011-04-13

**Authors:** Rui André Saraiva Raposo, Benjamin Thomas, Gabriela Ridlova, William James

**Affiliations:** 1 Sir William Dunn School of Pathology, University of Oxford, Oxford, United Kingdom; 2 Graduate Program in Areas of Basic and Applied Biology (GABBA), University of Porto, Porto, Portugal; 3 Central Proteomics Facility, Sir William Dunn School of Pathology, University of Oxford, Oxford, United Kingdom; The Research Institute for Children, United States of America

## Abstract

**Background:**

Human macrophages (Mφ) express low levels of CD4 glycoprotein, which is constitutively recycled, and 40–50% of its localization is intracellular at steady-state. Although CD4-interacting proteins in lymphoid cells are well characterised, little is known about the CD4 protein interaction-network in human Mφ, which notably lack LCK, a Src family protein tyrosine kinase believed to stabilise CD4 at the surface of T cells. As CD4 is the main cellular receptor used by HIV-1, knowledge of its molecular interactions is important for the understanding of viral infection strategies.

**Methodology/Principal Findings:**

We performed large-scale anti-CD4 immunoprecipitations in human primary Mφ followed by high-resolution mass spectrometry analysis to elucidate the protein interaction-network involved in induced CD4 internalization and degradation. Proteomic analysis of CD4 co-immunoisolates in resting Mφ showed CD4 association with a range of proteins found in the cellular cortex, membrane rafts and components of clathrin-adaptor proteins, whereas in induced internalization and degradation CD4 is associated with components of specific signal transduction, transport and the proteasome.

**Conclusions/Significance:**

This is the first time that the anti-CD4 co-immunoprecipitation sub-proteome has been analysed in human primary Mφ. Our data have identified important Mφ cell surface CD4-interacting proteins, as well as regulatory proteins involved in internalization and degradation. The data give valuable insights into the molecular pathways involved in the regulation of CD4 expression in Mφ and provide candidates/targets for further biochemical studies.

## Introduction

Mass spectrometry (MS)-based identification of the components of purified protein complexes has become one of the most powerful and routinely used technologies for high-throughput detection of protein interactions [1], [2]. The study of protein interactions by MS for identification of components of protein complexes gives powerful insights into protein function, binding partners and cellular pathways [3], [4]. In most studies, proteins in a given complex are identified via MS analysis of in-gel tryptic digests of electrophoretically separated proteins of particular sub-cellular fractions (membranes, nuclei, intracellular compartments) or in co-immunoprecipitated complexes [5], [6], [7], [8].

CD4 is the main cellular receptor used by human immunodeficiency viruses HIV-1, HIV-2 and simian immunodeficiency virus [9], [10], [11]. It is a type I transmembrane glycoprotein of 55 kDa expressed on the surface of Regulatory and Helper subsets of T lymphocytes and interacts with MHC class-II carrying cells [12]. CD4 increases the avidity of the low affinity interactions between the peptide-MHC complex on antigen presenting cells and the T cell receptor on the lymphocyte, and its association with the intracellular protein tyrosine kinase LCK modulates signal transduction [13]. In humans and rats CD4 is also expressed on cells of the monocyte/Mφ lineage, although its function on these cells is poorly understood, and the protein expression levels are 10- to 20-fold less than in T cells [14], [15]. In lymphoid cells expressing LCK, 90% of CD4 is restricted to the cell surface and undergoes limited internalization [16]. Endocytosis of CD4 can occur, through clathrin-coated pits, when the cytoplasmic domain becomes serine phosphorylated, leading to its dissociation from LCK [17], [18], [19]. In myeloid cells, such as Mφ, which do not express LCK, CD4 is constitutively internalized and 40–50% is intracellular at steady-state [16]. The pathways by which CD4 is removed from the cell surface and the protein-network involved are poorly defined. Cell surface CD4 levels can be down-regulated by exposure to gangliosides [20], soluble HIV-1 gp120 [21], phorbol esters [17], [22] and during HIV-1 infection [23], [24]. Moreover, down-regulation of viral receptors is a common mechanism used by most retroviruses to avoid superinfection (multiple rounds of infection) and to promote viral release. HIV-1 Nef protein accelerates CD4 internalization and degradation in the lysosomes [25], and at the late stages of HIV-1 infection, CD4 can be targeted for proteasomal degradation by HIV-1 Vpu [26], [27], [28].

Most reports to date have analysed CD4 interaction complexes in lymphoid cell lines, revealing some of the well-known associating proteins, such as LCK, CD45, transferrin receptor (CD71), CD98, myosins, vimentin, tubulins, actins, annexin II and lymphocyte phosphatase associated phosphoprotein (LPAP) [29], [30], [31], [32]. However, little is known about how CD4 antigen is arranged at the surface of Mφ, which notably lack LCK expression.

In common with other laboratories we found that the kinetics of HIV-1 replication was modulated by the simultaneous presence of Mφ and T cells in different ratios and activation states [33], [34], [35]. Data from our laboratory reported that HIV-1 viral production was typically slower in infected cultures in which Mφ were co-cultured with activated T cells. More recently, we extended these observations and showed that activated T cells produce soluble factors that selectively induce the internalization and degradation of CD4 in primary Mφ, thus critically affecting HIV-1 entry in a process sensitive to the vacuolar ATPase inhibitor bafilomycin A1, and the proteasomal inhibitor, MG132 (Saraiva Raposo et al., manuscript under revision).

**Figure 1:**
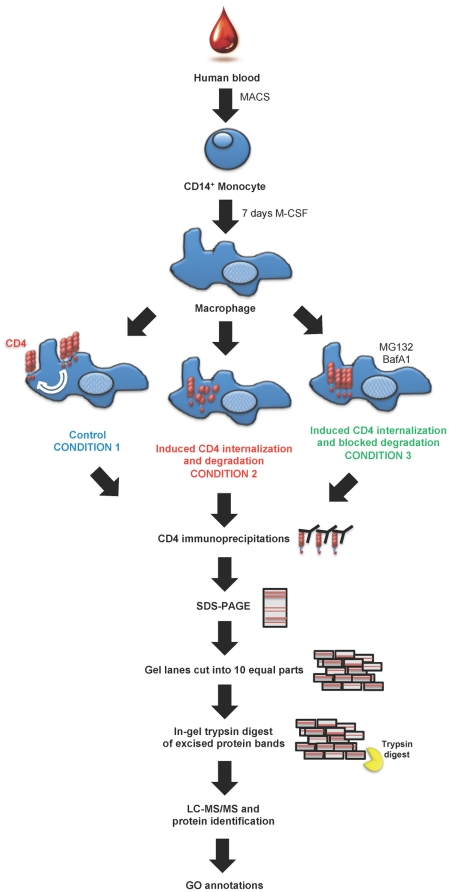
Strategy for the identification of CD4-complexes in human primary Mφ. CD14^+^ monocytes were isolated from human blood by magnetic cell sorting (MACS) and cultured for 7 days in the presence of M-CSF. One hundred million day 7 fully differentiated Mφ were left untreated (Condition 1, blue), treated with conditioned media from activated T cells (Induced CD4 internalization and degradation, Condition 2 red) or treated with conditioned media from activated T cells in the presence of the proteasomal inhibitor MG132 and the inhibitor of vacuolar ATPases bafilomycin (BafA1) (Induced CD4 internalization but blocked degradation, Condition 3 green). Eighteen hours later, cells were detached from tissue culture plates, lysed and large-scale anti-CD4 immunoprecipitations (IP) using monoclonal antibody against CD4 (clone QS4120) or isotype control IP were carried out. IP products were loaded onto SDS-PAGE pre-cast gels and electrophoresis were run. Protein gels were coomassie stained, gel lanes were cut into 10 equal pieces and trypsin-digested. Proteins were identified by LC-MS/MS.

In this report we perform high-resolution mass spectrometry analysis of CD4 co-immunoisolates in human primary Mφ, in order to characterise the CD4 containing complexes in steady-state and at different stages of CD4 internalization and degradation. The experimental strategy is shown in Fig. 1.

## Results

### Conditioned media from activated T cells induces CD4 internalization and degradation in Mφ

**Figure 2:**
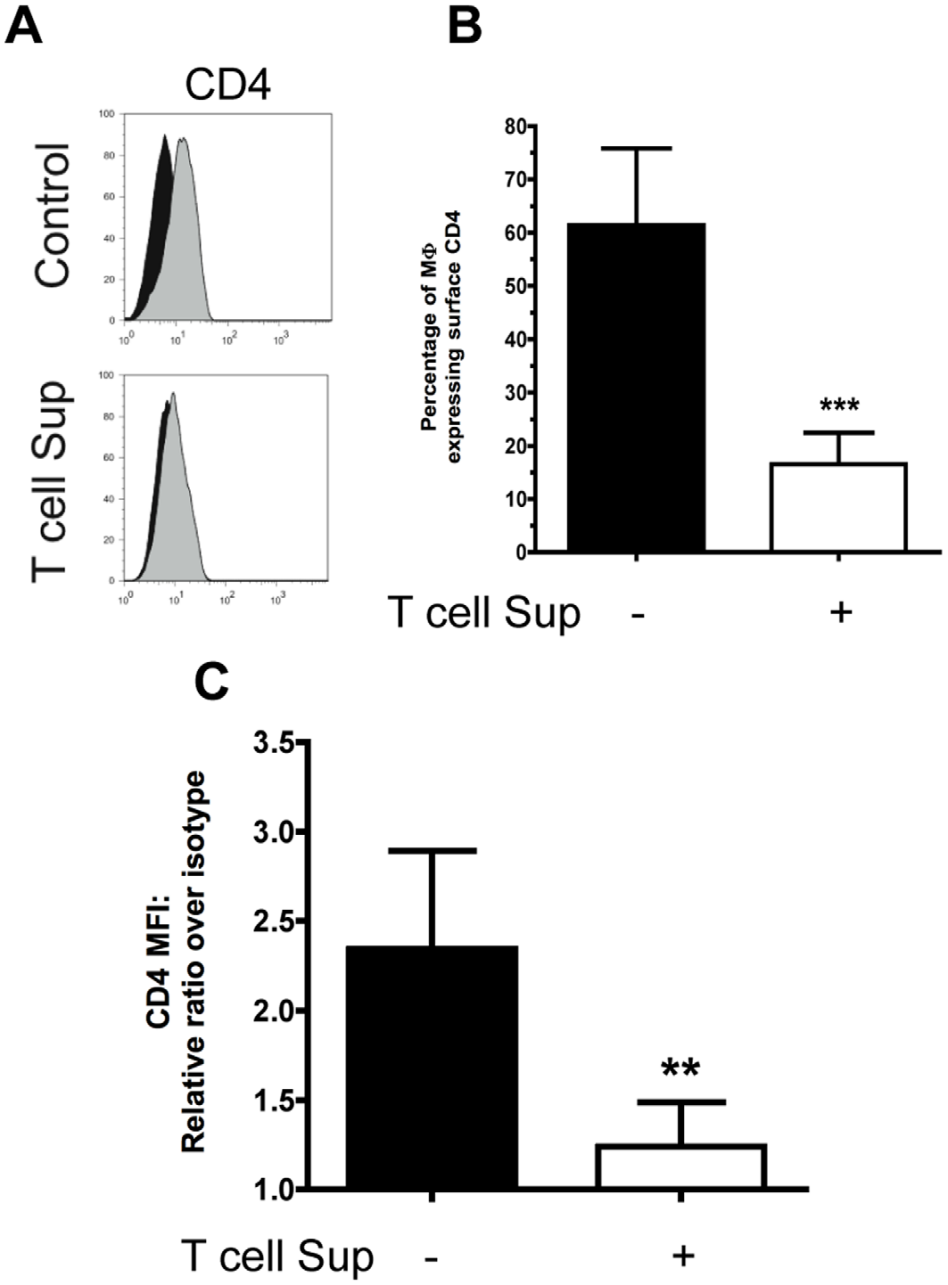
CD4 is internalized and degraded after treatment with conditioned media from activated T cells. Mφ were treated with conditioned media from activated T cells for 18 hours or left untreated, followed by flow cytometry staining with directly conjugated mAb to CD4. **A** Black histogram represents the appropriate isotype control. Histograms show the intensity of the signal on the X-axis with a log_10_-scale and the percentage of maximum expression on the Y-axis. Representative staining of more than five donors tested (n>5). **B** Bars represent the mean percentage of Mφ expressing surface CD4 with SD error bars from ten independent donors (n = 10). **C** Total CD4 expression levels (surface + intracellular) were determined by dividing the geometrical MFI of the antibody staining over the MFI of the isotype control. Bars represent the mean values of five independent donors (n = 5) with SD error bars. In **B** and **C**, black bar corresponds to untreated Mφ and white bar corresponds to conditioned media treated Mφ (T cell Sup).

In order to effectively demonstrate the induction of CD4 internalization and degradation, we detected the expression of CD4 in Mφ before and after treatment with conditioned media from activated T cells by flow cytometry. Eighteen hours post-treatment the expression of CD4 levels at the surface of Mφ was barely detectable (Fig. 2A), and the percentage of Mφ expressing surface CD4 was significantly reduced by 4-fold (Fig. 2B). In addition, total CD4 expression (surface + intracellular) was diminished by 2-fold (Fig. 2C). Altogether, these data suggest the internalization and degradation of CD4 after treatment with conditioned supernatants from activated T cells.

### Anti-CD4 co-immunoprecipitation sub-proteome in control Mφ

**Figure 3:**
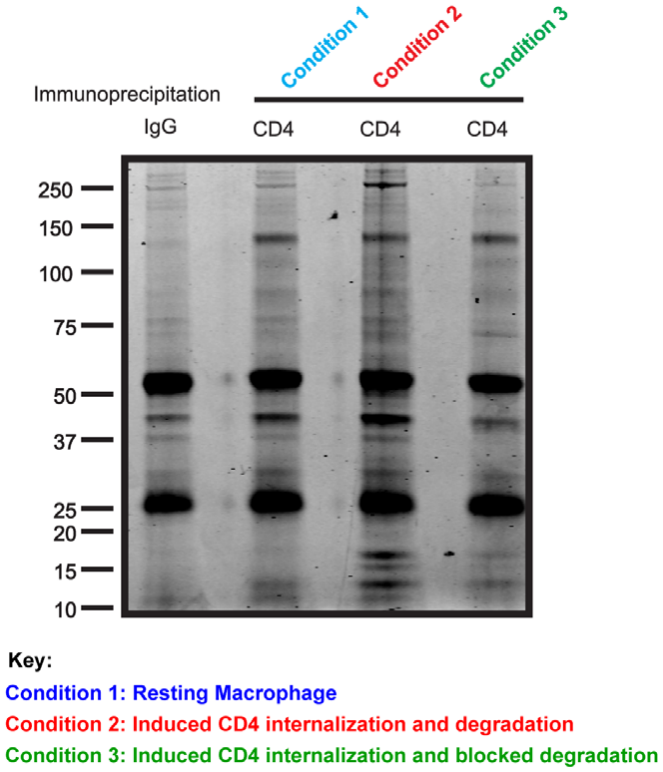
Representative protein gels of anti-CD4 immunoprecipitations in Mφ. Mφ were left untreated (Condition 1, blue), treated with conditioned media from activated T cells (Condition 2, red) or treated with conditioned media from activated T cells in the presence of 5 µM of MG132 and 100 nM of BafA1 (Condition 3, green). Eighteen hours later, cells were lysed and anti-CD4 immunoprecipitations were carried out. The final immunoisolates were resuspended in Laemmli sample buffer under reducing and denaturing conditions, before loading onto a SDS-PAGE pre-cast gel. Isotype control IgG immunoprecipitations were also performed to show non-specific background binding proteins.

We performed large-scale CD4 immunoprecipitations in normal resting primary human Mφ, followed by LC-MS/MS. A representative gel of the resolved proteins after CD4 co-immunoisolation is shown in Fig. 3. In control resting Mφ (condition 1), several cell surface proteins associated with CD4 were identified, including CD9, a tetraspanin-family member involved in cell adhesion, cell motility and IL-16 signalling [36], [37], [38], [39]; CD163, involved in the clearance and endocytosis of hemoglobin/haptoglobin complexes [40], [41]; integrin subunit beta (CD18), involved in cell surface adhesion and reported to interact with integrins alpha-M and alpha-X [42]; protein S100, a calcium binding protein known to be involved in phagocyte migration and infiltration at sites of wounding [43]; chemokine receptor 1 (CCR-1), a G protein-coupled receptor [44]; adaptor protein 2 (AP-2), a known adaptor protein which functions in protein transport via transport vesicles in different membrane trafficking pathways [25], [45], and HLA class I, involved in antigen presentation [46]. CD4 was also found to be associated with cytoskeleton and actin-modulating proteins, such as gelsolin, tropomyosins and dynein. An unknown and uncharacterised protein, TPP1 was also identified. A summary list of interacting proteins is shown in table 1.

**Table 1:** Uniquely identified proteins in anti-CD4 co-immunoprecipitations in control Mφ (Condition 1).

PROTEIN NAME	GENE	MOLECULAR WEIGHT	LOCALIZATION	FUNCTION/STRUCTURE	UNIPROT ACCESSION	PROBABILITY	UNIQUE PEPTIDES
Gelsolin, isoform 2	GSN	80,641	Cytoskeleton	Actin-modulating protein	P06396	1	14
Tropomyosin alpha-3 chain, isoform 2	TPM3	29,033	Cytoskeleton	Actin-modulating protein	P06753	1	6
Integrin beta 2	ITGB2	84,782	Membrane	Cell adhesion	P05107	1	5
Golgi autoantigen (Golgin), subfamily A2	GOLGA2	113,086	Golgi	cis-Golgi structure	Q08379	1	4
Tropomyosin alpha 4 chain, isoform 1	TPM4	28,522	Cytoskeleton	Actin-modulating protein	P67936	1	4
Putative uncharacterized protein TPP1	TPP1	60,369	Unknown	Unknown	B5MDC1	1	4
Coatomer, subunit gamma	COPG	97,718	Cytoplasm	Protein transport	Q9Y678	1	3
Cytoplasmic dynein 1, heavy chain 1	DYNC1H1	532,408	Microtubules	Motor protein	Q14204	1	3
Hematopoietic lineage cell-specific protein	HCLS1	53,984	Membrane	Antigen receptor signalling	P14317	1	3
AP-2 complex subunit beta, isoform 1	AP2B1	104,553	Membrane	Protein transport	P63010	1	3
Protein S100-A9	S100A9	13,242	Membrane	Chemotaxis	P06702	1	3
Actin-related protein 2/3 complex, subunit 1B	ARPC1B	40,950	Cytoplasm	Actin binding	O15143	1	2
Actin-related protein 2/3 complex, subunit 4	ARPC4	19,667	Cytoplasm	Actin binding	P59998	1	2
F-actin capping protein, subunit beta	CAPZB	37,482	Cytoplasm	Actin binding	B4DWA6	1	2
Scavenger receptor (M130) cysteine-rich	CD163	125,437	Membrane	Scavenger-receptor activity	Q86VB7	1	2
HLA class I histocompatibility antigen	HLA-C	36,798	Membrane	Antigen presentation	Q29960	0.9998	2
Protein S100-A8	S100A8	10,835	Membrane	Chemotaxis	P05109	1	2
Ras-related C3 botulinum toxin substrate 2	RAC2	21,429	Cytoplasm	GTP binding	P15153	1	2
Tropomyosin 1 alpha chain, isoform 2	TPM1	32,678	Cytoskeleton	Actin-modulating protein	Q9Y427	0.9996	2
C-C chemokine receptor type 1	CCR1	41,173	Membrane	G-protein coupled receptor protein	P32246	0.9888	2
CD9 antigen	CD9	25,416	Membrane	Signalling	P21926	0.9952	2

Protein and gene names, molecular weight in Daltons, cellular localization, function/structure, Uniprot accession number, protein identification probability from iProphet and unique number of identified peptides for each individual protein are shown.

### Anti-CD4 co-immunoprecipitation sub-proteome in induced internalization and degradation

Internalization and degradation of CD4 in Mφ was induced by conditioned media from activated T cells (condition 2) and interacting proteins were identified by CD4 co-immunoprecipitation followed by LC-MS/MS. A representative gel of the resolved proteins after CD4 co-immunoisolation is shown in Fig. 3. Proteins identified included Cdc42, a small GTPase family protein involved in signal transduction and endocytosis [47], [48]; proteins associated with late endocytic trafficking, such as LAMP1, a component of the lysosomal membrane [49], [50]; RhoB, known to be associated with the late endosome membrane; adaptor protein 1 (AP-1), a subunit of clathrin-associated adaptor protein complex 1 [45], [51], [52]; Sec23B, a component of coating protein II (COPII) involved in the transport of vesicles from the Golgi apparatus to the endoplasmic reticulum, and Rab10/Rab11B, important components of vesicle recycling and protein turn-over [45], [53]. Several cytoplasmic and cytoskeleton-related proteins were also identified, including fascin, myosin and tensin. Annexin A2, a calcium regulated membrane binding protein and flotillin-1, a scaffolding protein associated with caveolar membranes [54] were also identified with more than 5 unique peptides. A complete list of the uniquely identified proteins is shown in table 2.

**Table 2:** Uniquely identified proteins in anti-CD4 co-immunoprecipitations in induced CD4 internalization and degradation in Mφ (Condition 2).

PROTEIN NAME	GENE	MOLECULAR WEIGHT	LOCALIZATION	FUNCTION/STRUCTURE	UNIPROT ACCESSION	PROBABILITY	UNIQUE PEPTIDES
Actin, cytoplasmic 2	ACTG1	41,793	Cytoskeleton	Actin binding	P63261	0.9993	11
Annexin A2, isoform 1	ANXA2	38,604	Membrane	Calcium binding	P07355	1	9
Alpha actinin 4	ACTN4	104,854	Cytoplasm	Transport	O43707	1	6
Flotillin 1	FLOT1	47,355	Membrane	Protein transport	O75955	0.99775	6
Protein transport protein, Sec23B	SEC23B	86,479	COPII Vesicle	Protein transport	Q15437	1	5
Integrin beta	ITGB2	78,345	Membrane	Cell adhesion	A8MYE6	0.99825	3
Fascin	FSCN1	54,530	Cytoplasm	Actin binding	Q16658	1	2
Myosin-Va, isoform 1	MYO5A	215,405	Cytoplasm	Actin binding	Q9Y4I1	1	2
Tensin 3, isoform 1	TNS3	155,266	Cytoplasm	Protein binding	Q68CZ2	0.9955	2
Cytosolic non-specific dipeptidase, isoform 2	CNDP2	43,833	Cytoplasm	Proteolysis	Q96KP4	1	2
Reticulon 4, isoform 2	RTN4	40,318	Membrane	Protein binding	Q9NQC3	1	2
Ras-related protein, Rab-10	RAB10	22,541	Membrane	Protein transport	P61026	0.99775	2
Ribonuclease inhibitor	RNH1	49,973	Cytoplasm	Protein binding	P13489	1	2
Cell division control protein 42, Isoform 1	CDC42	21,311	Cytoplasm/Membrane	GTP binding	P60953	0.9955	2
AP-1 complex subunit beta 1, Isoform A	AP1B1	104,637	Clathrin Coated Pits	Endocytosis	Q10567	0.9955	2
Lysosome associated membrane glycoprotein 1	LAMP1	44,882	Lysosome	Protein degradation	P11279	0.9955	2
Ras-related protein, Rab-11B	RAB11B	24,489	Membrane	Protein transport	Q15907	0.9965	2
Rho-related GTP-binding protein, RhoB	RHOB	22,123	Membrane	Protein transport	P62745	0.9876	2

Protein and gene names, molecular weight in Daltons, cellular localization, function/structure, Uniprot accession number, protein identification probability from iProphet and unique number of identified peptides for each individual protein are shown.

### Anti-CD4 co-immunoprecipitation sub-proteome in induced internalization and blocked degradation

In condition 3, internalization of CD4 in Mφ was induced by the same conditioned media from activated T cells, as described for condition 2, and cellular degradation was blocked using the proteasome inhibitor MG132 and the vacuolar ATPase inhibitor bafilomycin A1. CD4-interacting proteins were identified by co-immunoprecipitations followed by LC-MS/MS. A representative gel of the resolved proteins after CD4 co-immunoisolation is shown in Fig. 3. CD4 was associated with a large number of proteins related to protein degradation, in particular the proteasome. Proteasome-related proteins such as the 26S regulatory subunit 6B, ubiquitin-like modifier activating enzymes E1 and E3 ubiquitin protein ligase subunit Itch [55], [56], [57], [58] were identified. Proteins associated with antigenic presentation and intracellular protein trafficking were also identified, such as MHC-I molecules (HLA-A and HLA-B), ERp29 and ERp1 (endoplasmic reticulum chaperones) [59]. Although identified with one unique peptide, but with high iProphet probability scores, we also detected 7 proteins, including components of vacuolar proton-transporting ATPases, such as V-type proton ATPase subunits D and G1. A complete list of the uniquely identified proteins is shown in table 3.

**Table 3:** Uniquely identified proteins in anti-CD4 co-immunoprecipitations in induced CD4 internalization and blocked degradation in Mφ (Condition 3).

PROTEIN NAME	GENE	MOLECULAR WEIGHT	LOCALIZATION	FUNCTION/STRUCTURE	UNIPROT ACCESSION	PROBABILITY	UNIQUE PEPTIDES
Heat shock 70 kDa protein 1/2	HSPA1B	70,052	Cytoplasm	Chaperone, protein folding	P08107	1	19
Coronin 1C	CORO1C	49,379	Cytoskeleton	Signal transduction	B4DMH3	1	3
Heme oxygenase 1	HMOX1	32,819	ER	Metal-binding	P09601	1	3
Guanine nucleotide-binding protein G, isoform 2	GNAI2	38,473	Membrane	GTP Binding, signal Transduction	P04899	1	3
Annexin IV	ANXA4	36,085	Cytoplasm	Calcium binding	Q6LES2	1	2
Annexin VI	ANXA6	75,277	Cytoplasm	Calcium binding	A6NN80	0.7873	2
Endoplasmic reticulum protein, ERp29	ERP29	28,993	ER lumen	Intracellular protein transport	P30040	1	2
Guanine nucleotide-binding protein subunit beta 4	GNB4	37,567	Cytoplasm	Transmembrane signalling	Q9HAV0	0.9989	2
HLA class I histocompatibility antigen	HLA-A	40,892	Membrane	Antigen processing and presentation	P16190	1	2
Hypoxia up-regulated protein 1	HYOU1	111,335	ER lumen	Chaperone, protein folding	Q9Y4L1	1	2
E3 ubiquitin-protein ligase Itchy, isoform 1	ITCH	102,803	Cytoplasm	Protein ubiquitination	Q96J02	1	2
Heterogeneous nuclear ribonucleoprotein R	HNRNPR	70,943	Cytoplasm	mRNA processing	O43390	0.9931	2
Ras-related protein Rab-1A	RAB1A	22,678	Membrane	Protein transport	P62820	0.9898	2
Endoplasmic reticulum aminopeptidase 1, isoform 2	ERAP1	107,841	ER lumen	Antigen processing and presentation	Q9NZ08	1	2
Ras-related protein, Rab-1B	RAB1B	22,171	Membrane	Protein transport	Q9H0U4	0.9898	2
26S protease regulatory subunit 6B	PSMC4	47,366	Proteasome Complex	Protein degradation	P43686	0.9971	2
Proteasome activator complex, subunit 1	PSME1	28,723	Proteasome Complex	Protein degradation	Q06323	0.9971	2
Proteasome subunit alpha type 4	PSMA4	29,484	Proteasome Complex	Protein degradation	P25789	0.9971	2
Ubiquitin-like modifier-activating enzyme 1	UBA1	117,849	Cytosol	Ubiquitin conjugation pathway	P22314	0.9971	2
Antigen peptide transporter 1	TAP1	87,218	ER lumen	Protein transport	Q03518	0.9971	1
HLA class I histocompatibility antigen	HLA-B	40,481	Membrane	Antigen processing and presentation	P30481	0.9971	1
Tyrosine-protein phosphatase non-receptor	PTPN6	67,561	Cytoplasm	Signal transduction	P29350	0.9778	1
Ras-related protein, Rab-14	RAB14	23,897	Membrane	Protein transport	P61106	0.9971	1
Transmembrane emp24 domain-containing protein	TMED10	24,976	Golgi apparatus membrane	Vesicular protein trafficking	P49755	0.9971	1
V-type proton ATPase subunit D	ATP6V1D	28,263	Vacuole	Proton-transporting ATPase	Q9Y5K8	0.9971	1
V-type proton ATPase subunit G1	ATP6V1G1	13,758	Vacuole	Proton-transporting ATPase	O75348	0.9969	1

Protein and gene names, molecular weight in Daltons, cellular localization, function/structure, Uniprot accession number, protein identification probability from iProphet and unique number of identified peptides for each individual protein are shown.

Table 4 lists the proteins commonly identified in all three conditions.

**Table 4:** Proteins commonly identified in all conditions.

PROTEIN NAME	GENE	MOLECULAR WEIGHT	LOCALIZATION	FUNCTION/STRUCTURE	UNIPROT ACCESSION	PROBABILITY	UNIQUE PEPTIDES
Myosin 9, isoform 1	MYH9	226,532	Cytoplasm	Actin binding	P35579	1	126
Ras GTPase-activating-like protein	IQGAP1	189,252	Membrane	Ras GTPase activator activity	P46940	0.99954	40
Major vault protein	MVP	99,327	Cytoplasm	Protein transport	Q14764	1	35
Filamin A, isoform 2	FLNA	280,018	Cytoskeleton	Protein binding	P21333	1	34
Vimentin	VIM	53,652	Cytosol	Actin binding	P08670	1	28
Plastin 2	LCP1	70,289	Cytoplasm	Actin binding	P13796	1	22
Clathrin heavy chain 1	CLTC	191,615	Clathrin coated pit	Protein transport	Q00610	1	20
Protein transport protein, Sec16A	SEC16A	233,517	ER/Golgi	Protein transport	O15027	0.99478	19
Endoplasmin	HSP90B1	92,469	Cytosol	ERAD protein catabolism	P14625	0.99998	13
Alpha actinin 1	ACTN1	103,058	Cytoskeleton	Actin binding	P12814	1	11
Talin 1	TLN1	269,767	Cytoskeleton	Actin binding	Q9Y490	0.9996	11
Moesin	MSN	67,820	Cytoskeleton	Cell adhesion	P26038	1	10
DnaJ subfamily C member 10	DNAJC10	91,080	ER lumen	Protein folding	Q8IXB1	0.9977	9
CD4 antigen	CD4	51,111	Membrane	Receptor activity	P01730	0.99942	9
Protein transport protein, Sec24C	SEC24C	118,325	COPII vesicle	ER/Golgi transport	P53992	0.95185	8
Annexin A5	ANXA5	35,937	Cytoplasm	Calcium binding	P08758	0.9988	7
Profilin 1	PFN1	15,054	Cytoskeleton	Actin binding	P07737	0.99954	7
Protein disulfide isomerase	P4HB	57,116	Membrane	Protein disulfide isomerase	P07237	0.99735	6
V-type proton ATPase, subunit B	ATP6V1B2	56,501	Cytosol	Proton-transporting ATPase	P21281	0.99883	6
Calreticulin	CALR	48,142	Cytosol	Calcium binding	P27797	0.9984	5
Cathepsin B	CTSB	37,822	Lysosome	Degradation/turn-over of proteins	P07858	0.99787	5
Coronin 1A	CORO1A	51,026	Cytoskeleton	Actin binding	P31146	0.99748	5
Cofilin 1	CFL1	18,502	Cytoskeleton	Actin binding	P23528	0.9987	4
F-actin-capping protein, subunit alpha 2	CAPZA2	32,949	Cytoskeleton	Actin binding	P47755	0.9966	4
Protein transport protein, Sec24A	SEC24A	119,749	COPII Vesicle	ER/Golgi transport	O95486	0.99903	4
Myeloid cell nuclear differentiation antigen	MNDA	45,836	Cytoplasm	Transcription regulation	P41218	0.99806	4
Transferrin receptor protein 1	TFRC	84,871	Membrane	Transferrin receptor activity	P02786	0.9967	4
14-3-3 protein zeta/delta	YWHAZ	27,745	Cytosol	Signal transduction	P63104	0.99913	3
Integrin alpha M	CD11b	127,179	Membrane	Cell adhesion	P11215	0.99747	3
Protein-glutamine gamma-glutamyltransferase 2	TGM2	77,329	Membrane	Cell adhesion	P21980	0.99808	3
Lymphocyte-specific protein 1	LSP1	37,192	Membrane	Signal transduction	P33241	0.99903	3
Macrophage-capping protein	CAPG	38,518	Cytosol	Actin binding	P40121	0.99758	3
Ras-related protein, Rap-1b	RAP1B	20,825	Membrane	GTPase activity	P61224	0.99743	3
IgE Fc receptor subunit gamma	FCER1G	9,667	Membrane	Receptor activity	P30273	0.99773	2
Protein S100-A11	S100A11	11,740	Cytosol	Calcium binding	P31949	0.99883	2

Protein and gene names, molecular weight in daltons, cellular localization, function/structure, Uniprot accession number, protein identification probability from iProphet and unique number of identified peptides for each individual protein are shown.

### Western Blotting analysis of CD4 co-immunoprecipitates in Mφ

**Figure 4:**
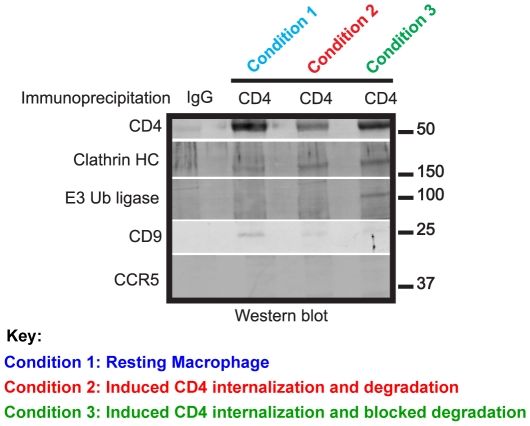
Western blot analysis of CD4 co-immunoprecipitates in Mφ. A total of 1×10^7^ Mφ were left untreated (Condition 1, blue), treated for 18 hours with supernatants from activated T cells (Condition 2, red), treated for 18 hours with supernatants from activated T cells in the presence of 5 µM MG132 and 100 nM BafA1 (Condition 3, green), lysed and anti-CD4 immunoprecipitation reactions were carried out. Isotype control immunoprecipitations were also performed to show background protein binding. Immunoisolates were resuspended in Laemmli sample buffer under reducing and denaturing conditions and resolved on a SDS-PAGE gel. Membranes were incubated with antibodies against CD4, clathrin heavy chain (HC) 1, E3 Ubiquitin (Ub) ligase Itch, CD9 and CCR5. Primary antibodies were detected and scanned using the quantitative western blotting imaging Odyssey System. A representative blot of three different blood donors is shown (n = 3).

Mass spectrometry identifications of CD9, E3 ubiquitin ligase Itch and clathrin heavy chain in CD4 co-immunoisolates were confirmed by western blot analysis. As anticipated, CD4 was identified in all Mφ sample conditions, but at reduced levels in condition 2. Clathrin heavy chain 1 was co-immunoisolated with CD4 in all three conditions and the E3 ubiquitin ligase subunit Itch was only co-immunoisolated with CD4 when cellular degradation was blocked. CD9 antigen was only co-immunoisolated with CD4 in the control Mφ. CCR5, reported to interact with CD4 at the surface of Mφ and T cells [60], was not identified by mass spectrometry in any of the conditions described above and was not detected by western blot analysis of CD4 co-immunoisolates (Fig. 4).

### GO annotations

**Figure 5:**
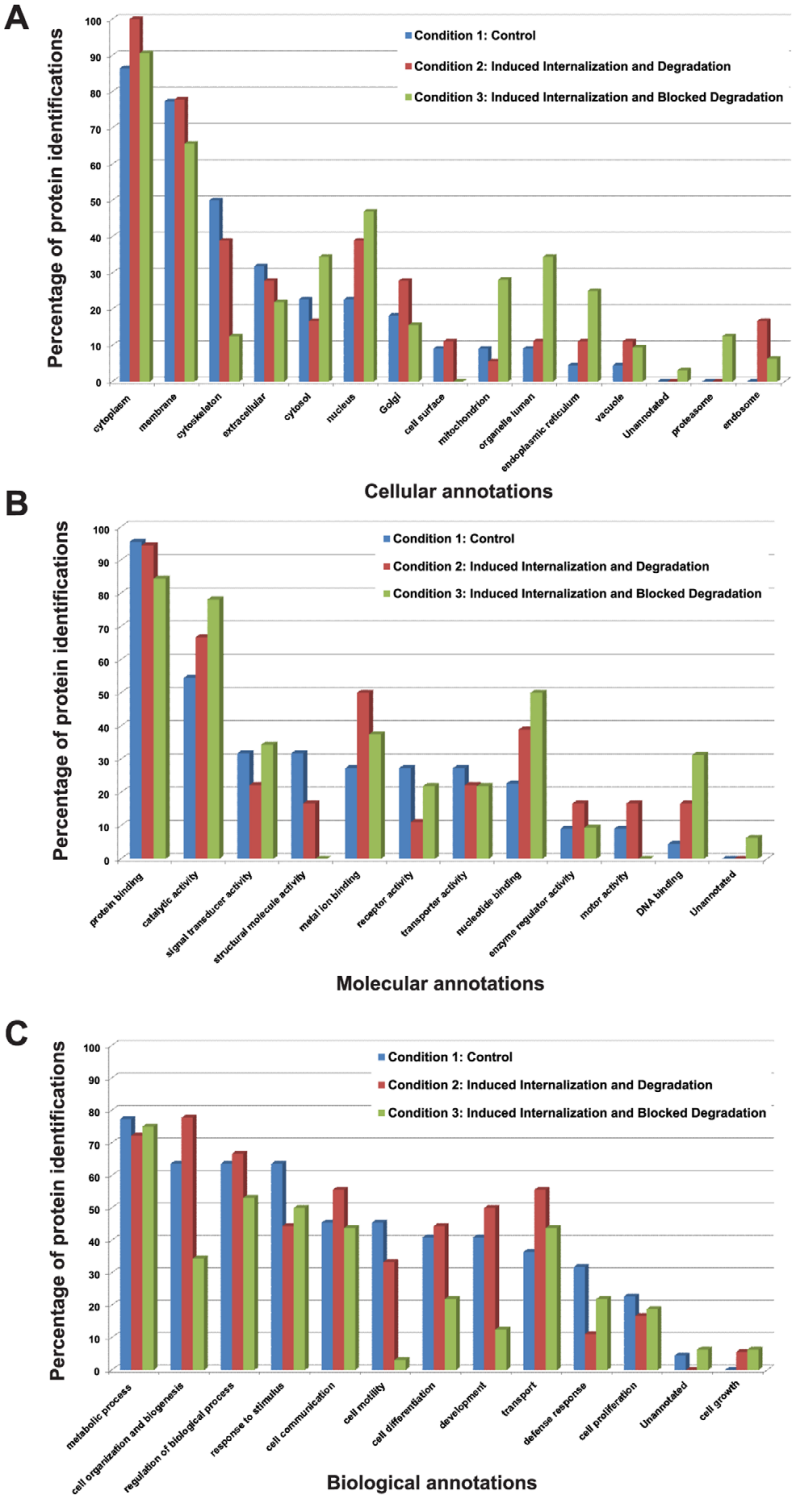
Gene Ontology (GO) annotations of the uniquely identified proteins in anti-CD4 immunoprecipitations in Mφ. Protein identifications from the three different conditions were exported from the in-house developed Central Proteomics Facilities data analysis pipeline (CPFP) and uploaded to ProteinCenter software. **A** illustrates the percentage of protein identifications versus protein cellular localizations (GO cellular annotations); **B** illustrates the percentage of protein identifications versus protein molecular functions (GO molecular annotations) and **C** illustrates the percentage of protein identifications versus protein biological functions (GO biological annotations). Blue bars represent the percentage of unique proteins identified in condition 1 (Resting macrophages); Red bars represent the percentage of unique proteins identified in condition 2 (Induced CD4 internalization and degradation); Green bars represent the percentage of unique proteins identified in condition 3 (Induced CD4 internalization and blocked degradation).

Uniquely identified protein identifications in all three conditions were exported to ProteinCenter and GO annotations were carried out. In induced CD4 internalization and degradation (condition 2) there is an over-representation of proteins associated with the endosome, vacuole and Golgi, when compared to control Mφ (condition 1). Moreover, when cellular degradation is blocked (condition 3) the over-represented CD4-associated proteins are related to the proteasome, endoplasmic reticulum, organelle lumen, mitochondrion and cytosol (Fig. 5A). Proteins related to DNA and nucleotide-binding are over-represented in condition 3 and metal binding proteins are over-represented in condition 2. No proteins with structural molecular activities were uniquely identified in condition 3, in contrast to control or condition 2, where 30% and 15%, respectively, of the uniquely identified proteins fall into this category (Fig. 5B). Proteins related to cell organization and biogenesis, cell differentiation, development and transport are greatly over-represented in condition 2 over condition 3. In control Mφ, proteins related to response to stimulus and defence response are over-represented over the other two. Cell motility-related proteins cluster with CD4 in control Mφ and in condition 2 (Fig. 5C).

## Discussion

Mass spectrometry analysis of CD4 co-immunoisolates, supplemented with GO annotations provided useful information on the clustering of CD4 molecules in resting Mφ and elucidated the protein-network involved in the internalization and degradation. CD4 in resting Mφ showed association with a range of molecules found in the cellular cortex and membrane rafts. Consistent with earlier reports [19], [25], [61], we also observed CD4 association, and confirmed by western blotting, with components of clathrin-mediated endocytosis, such as clathrin heavy chain 1 and the adaptor protein AP-2, clearly suggesting that in resting Mφ CD4 undergoes constitutive internalization and recycling [16], [18], [62]. AP-2 has been reported to be involved in the initial formation of clathrin coated pits at the plasma membrane, and it is an important mediator of receptor internalization and clathrin assembly [63]. We observed CD4 association with the tetraspanin protein CD9, and as both CD4 and CD9 are able to bind IL-16 in mast cells [36], [64], this association might in fact be physiologically relevant in Mφ.

In addition to CD4, HIV-1 requires CXCR4 or CCR5 to enter target cells. Xiao et al., reported a constitutive cell surface association between CD4 and CCR5 [60] and showed that the presence of gp120, leads to the clustering of CD4 and CCR5. However, they stated that it was difficult to co-immunoisolate CD4 and CCR5 in human primary Mφ and CD4^+^ T cells in the absence of gp120, arguing that the levels of both receptors were very low and the techniques used were not sensitive enough. Employing high-resolution mass spectrometry analysis on a large sample of primary Mφ, a more sensitive technique than the one used by Xiao et al., we did not detect CCR5 molecules in CD4 co-immunoisolates. Although a constitutive CD4-CCR5 interaction in the absence of gp120 might still exist, our results do not support this notion.

Many reports to date have shown that in CD4^+^ T cells LCK binds directly to the cytoplasmic tail of CD4 [13], [16], [18], providing stability at the cell surface. As we did not identify any Src family protein kinases in CD4 co-immunoisolates in Mφ, it seems unlikely that this kinase family plays a similarly prominent role in the regulation of CD4 in Mφ, as it does in T cells. This could also explain the faster turn-over of CD4 in Mφ compared to T cells.

Data from our laboratory showed that upon treatment with conditioned media from activated T cells, CD4 expression in Mφ is down-regulated due to induced internalization and degradation (Saraiva Raposo et al., manuscript under revision). Under this condition, CD4 was associated with specific components of signal transduction and transport pathways, including plasma membrane-associated small GTPases, such as Cdc42, Ras-related proteins and RhoB. The small GTPases of the Ras superfamily are well known to have roles in endocytosis [65], [66]. RhoB regulates endosomal trafficking, in co-operation with mDia1 and Src kinase [67], and Cdc42, which has also been connected to cell migration and cell polarity, has also been linked to the regulation of endocytosis [68]. We observed an interaction between CD4 and LAMP1, suggesting the intervention of lysosomes in the down-regulation of CD4. This observation correlates with the effect induced by the phorbol ester PMA in the induction of CD4 internalization and degradation [69]. Overall, the over-representation of endosome-related proteins in this condition, clearly clusters CD4 with the endocytic pathways.

When Mφ are treated with conditioned media from activated T cells in the presence of MG132 and bafilomycin A1, CD4 can still be internalized, but it is not degraded (Saraiva Raposo et al. manuscript under revision). Under this condition, CD4 was associated with several components of the proteasome, such as regulatory and activating subunits involved in the cascade of protein ubiquitination, suggesting the involvement of the proteasomal pathway. We identified the member of the E3 ubiquitin (Ub) ligase family, Itch/AIP4 to be associated with CD4 and confirmed it by western blot. Itch is a member of the HECT domain-containing E3 Ub ligases and has been implicated in the post-translational modification with Ub of CXCR4, followed by desensitization at the cell surface by engagement to its cognate ligand SDF-1α [70].

In the early stages of HIV-1 infection, the viral protein HIV-1 Nef, reported to accelerate CD4 down-regulation, avoiding viral superinfection and promoting efficient viral spread and optimal viral particle production [25], also alters the intracellular trafficking of MHC-I and MHC-II molecules [71]. HIV-1 Nef-dependent reduction of surface MHC-I protects HIV-infected primary T cells from recognition and killing by HIV-specific cytotoxic T cells in vitro [72]. Schaefer et al. reported that HIV-1 Nef targets MHC-I molecules and CD4 for degradation in the lysosomes, by showing co-localization of CD4 and a subset of HLA-A2 proteins in late endosomes and multi-vesicular bodies (MVB) [73]. We showed an interaction between CD4 and components of MHC-I (HLA-A and HLA-B). Although, our system is an HIV-1 Nef-independent system, both induced pathways seem to have some degree of similarity.

Overall in resting macrophages CD4 shows association with a range of proteins found in the cellular cortex, clathrin coated pits and membrane rafts. In induced internalization the spectrum of proteins clustered with the receptor changes and CD4 becomes associated with components of signal transduction and transport. Finally, under conditions where protein degradation pathways are chemically blocked, CD4 associates with components of the proteasome and ubiquitin-modifying proteins.

This is the first co-immunoisolation LC-MS/MS-based identification of CD4 complexes in human primary Mφ elucidating CD4-interacting proteins and the protein-network involved in its induced internalization and degradation. Due to its importance in the context of HIV-1 infection, revealing the CD4 “interactome” can lead to the discovery of important proteins in the pathogenesis of the virus. In conclusion, our mass spectrometry data contribute to a better understanding of the fate of CD4 molecules in resting Mφ and in induced internalization and degradation.

## Materials and Methods

### Ethics statement

Adult human blood was obtained from anonymous donors through the UK National Blood Service and tested negative for HIV-1, hepatitis B/C, and syphilis. Local IRB approval was sought for this work from Oxford University's Central University Research Ethics Committee (CUREC), and we were informed that specific ethical approval was unnecessary for this study, in accordance with their guidelines on the use of human blood (http://www.admin.ox.ac.uk/curec/resrchapp/faqethapp.shtml): “CUREC does not require an ethics form for laboratory research using buffy coats. However there are occasions when the National Blood Service donating the buffy coats may require ethical approval from the University. In this instance a checklist completion will suffice. Applicants should answer Question C (8) as a ‘NO’. A covering note should be sent to the Secretary of the MSD IDREC with the checklist explaining that the research uses buffy coats and the NBS requires University ethical approval.” Although not required by NBS, we completed a checklist as indicated and received exemption from MSD IREC.

### Cells and reagents

PBMC were isolated using Ficoll-Plaque Plus (GE Healthcare Life Sciences, Europe) density gradient centrifugation from heparinized buffy-coats. Monocytes were isolated by CD14-positive selection using anti-CD14 magnetic beads (Miltenyi Biotec, UK), according to the manufacturer's instructions and seeded in complete medium (RPMI 10% FCS (PAA), 2 mM L-glutamine (PAA), 100 U/mL penicillin (PAA) and 100 µg/mL streptomycin (PAA)), supplemented with 50 ng/mL recombinant M-CSF (R&D Systems) for 7 days. MG132 and bafilomycin A1 (BafA1) (Sigma, UK) were resuspended in DMSO (Sigma, UK) and used at final non-toxic concentrations of 5 µM and 100 nM, respectively. CD4^+^ T Helper cells were isolated from the CD14-negative population of PBMC, by negative selection (Miltenyi Biotec, UK), according to the manufacturer's protocol and activated using anti-biotin MACSiBead particles and biotinylated antibodies against human anti-CD2, CD3 and CD28 (Miltenyi Biotec, UK) in complete medium for 3 days. Cell-free supernatants were collected after 3 days stimulation, filtered (0.45 µm pore-size) and stored until used. Typically, day 7 fully differentiated Mφ were treated with neat T cell supernatants, in the absent or presence of MG132 and BafA1 for 18 hours, prior to CD4 co-immunoisolation.

### Flow cytometry

CD4 expression levels were detected by direct immunofluorescence. Mφ in staining buffer (10 µg/mL human IgG (Sigma UK), 1% FCS and 0.01% NaN_3_) were incubated with 5 µg/mL anti-CD4 specific mAb (clone RPA-T4, Becton Dickinson) or matched isotype control (IgG1κ, Becton Dickinson) on ice for 30–45 min. For intracellular staining, cells were first fixed, then permeabilized with 0.2% saponin (Sigma, UK) and stained. The percentage of positive cells and the mean fluorescence intensity (MFI) were analyzed by FACS Calibur (Becton Dickinson) with 15,000**–**20,000-gated events collected. The data was processed using FlowJo (version 7.2.4). Protein expression levels were determined by dividing the geometrical MFI of the Ab staining over the MFI of the isotype control.

### Western blotting

Adherent Mφ were washed free of media, detached using ice cold 10 mM EDTA/PBS and cell pellets were lysed in ice-cold lysis buffer (50 mM Tris-HCl pH 8, 150 mM NaCl, 1% (v/v) n-Dodecyl β-D-maltoside (Sigma), 1× protease inhibitor cocktail (Roche), phosphatase inhibitor cocktail 2 (Sigma)). n-Dodecyl β-D-maltoside is a water-soluble non-ionic detergent, shown to be a rather gentle detergent able to preserve protein activity and structure better than many commonly used agents, such as Triton X-100, NP-40, CHAPs and octyl-β-glucoside [74], [75], [76], [77]. Lysates were centrifuged for 10 min at 4°C, 13,000×g to separate insoluble material and cleared lysate was resuspended in 1× Laemmli sample buffer (Invitrogen, UK) under reducing conditions and heated for 10 min at 90°C. Lysates were electrophoresed through SDS–PAGE gels and proteins were electroblotted to PVDF transfer membranes. Blocked membranes were incubated with one of the following primary antibodies diluted in 3% (w/v) BSA (Sigma) in 1× PBS-T (1× PBS, 0.1% (v/v) Tween-20) for 2 hours at room temperature or over-night at 4°C: rabbit polyclonal antibody anti-CD4 (clone H-370), rabbit polyclonal antibody anti-CD9 (clone H-110), rabbit polyclonal antibody anti-clathrin heavy chain 1 (clone H-300), rabbit polyclonal antibody anti-E3 Ubiquitin ligase (clone H-110) (all from Santa Cruz) and mouse monoclonal antibody anti-CCR5 (clone CTC5, R&D Systems). Primary antibodies were detected using the matching LI-COR secondary antibodies and membranes were scanned using the quantitative western blotting imaging system Odyssey (LI-COR).

### Immunoisolation analysis

Anti-CD4 immunoisolation reactions consisted of 10 µL of protein G–Sepharose bead slurry (4B Fast Flow, Sigma, UK) per 1×10^7^ lysed cells and 5–10 µg mouse monoclonal antibody anti-CD4 (clone QS4120, Santa Cruz) was incubated for 2 hours at room temperature to allow binding of the antibody to the beads. Beads were gently spun, cell lysate was added to the mixture of beads/antibody and the reactions were incubated by inversion for 3 hours at 4°C. The immunoisolates were collected by centrifugation for 5 min at 4°C, and washed three times for 5 min with lysis buffer. The final immunoisolates were resuspended in Laemmli sample buffer under reducing conditions and heated for 10 min at 90°C, before loading them onto a gel. Isotype control immunoprecipitations were also performed to identify background binding proteins.

### Mass spectrometry and protein identification

Anti-CD4 or isotype control immunoisolated pellets were reduced in NuPAGE sample reducing agent (Invitrogen, UK), separated on a NuPAGE Novex 4–12% Bis-Tris gel (Invitrogen, UK) and coomassie stained. Gel lanes were excised, cut into 10 equal portions and in-gel digested with trypsin [78]. Briefly, gel bands were diced into cubes and destained in 25 mM ammonium bicarbonate in 50∶50 water/acetonitrile. Proteins were reduced with 10 mM DTT and alkylated with 55 mM iodoacetamide. Gel bands were then incubated with 3 µg of trypsin (Promega, UK) in 25 mM ammonium bicarbonate over-night at 37°C. Peptides were extracted and desalted using home-made C18 tips. Mass spectrometry data were acquired on an Orbitrap mass spectrometer (Thermo) fitted with a nanospray source (Proxeon, Denmark) coupled to a U3000 nano HPLC system (Dionex, UK). The samples were loaded onto a 15 cm long, 100 micron ID, home-packed column manufactured by packing a Picotip emitter (New Objective, USA) with ProntoSIL C18 phase; 120 angstrom pore, 3 micron bead, C18 (Bischoff Chromatography, Germany). HPLC was run in a direct injection configuration. One hundred and twenty minute gradients were used to resolve the peptides. The Orbitrap was run in a data dependent acquisition mode in which the Orbitrap resolution was set at 60,000 and the top 5 multiply charged precursors were selected for MS/MS fragmentation. Samples were typically injected three times in order to increase the number and confidence of identifications. RAW data files were converted to mzXML format using ReAdW (version 4.2.1) and submitted to the in-house developed Central Proteomics Facilities Pipeline (CPFP) [79]. The CPFP is based on the Trans Proteomic Pipeline tools (version 4.2.1) [80] and implements automatic identification of MS/MS spectra using multiple search engines to maximise coverage of a sample. mzXML files were converted to suitable peaklist formats for submission to Mascot (Matrix Science), X!Tandem with k-score plugin [81] and OMSSA [82]. Searches are performed automatically and executed on a compute cluster, using Sun GridEngine, and the resulting peptide identifications from each search engine are validated with PeptideProphet [83]. iProphet is used to combine peptide hits from each three search engines and refines identification probabilities. ProteinProphet infers protein identifications from the resulting combined peptide list and performs grouping of ambiguous hits [84]. Protein identifications were exported from the CPFP and uploaded to ProteinCenter (Proxeon, Denmark) for filtering, annotation, classification, and interpretation. Searches were performed against a concatenated target/decoy human IPI database providing an empirical false discovery rate (FDR) and criteria for protein identification included 1% FDR and two or more unique peptides identified for each individual protein. Proteins that were identified in the isotype control immunoprecipitations were filtered out of the final interpretation. Uniquely identified proteins were only identified in the condition tested and commonly identified proteins were identified in all conditions tested.

### Statistical analysis

Statistical analysis was performed by paired t-test using GraphPad Prism (version 5.01). Stars indicate the p-value: **p = 0.01-0.001; ***p<0.001. Significance refers to difference from the controls, unless otherwise indicated. N refers to the number of blood donors tested.
